# Biomimicking Extracellular Vesicles with Fully Artificial Ones: A Rational Design of EV-BIOMIMETICS toward Effective Theranostic Tools in Nanomedicine

**DOI:** 10.1021/acsbiomaterials.2c01025

**Published:** 2022-12-19

**Authors:** Giada Rosso, Valentina Cauda

**Affiliations:** Department of Applied Science and Technology, Politecnico di Torino, Corso Duca degli Abruzzi 24, 10129 Turin, Italy

**Keywords:** extracellular vesicles, artificial EV, theranositc nanoparticle, lipidomics, proteomics

## Abstract

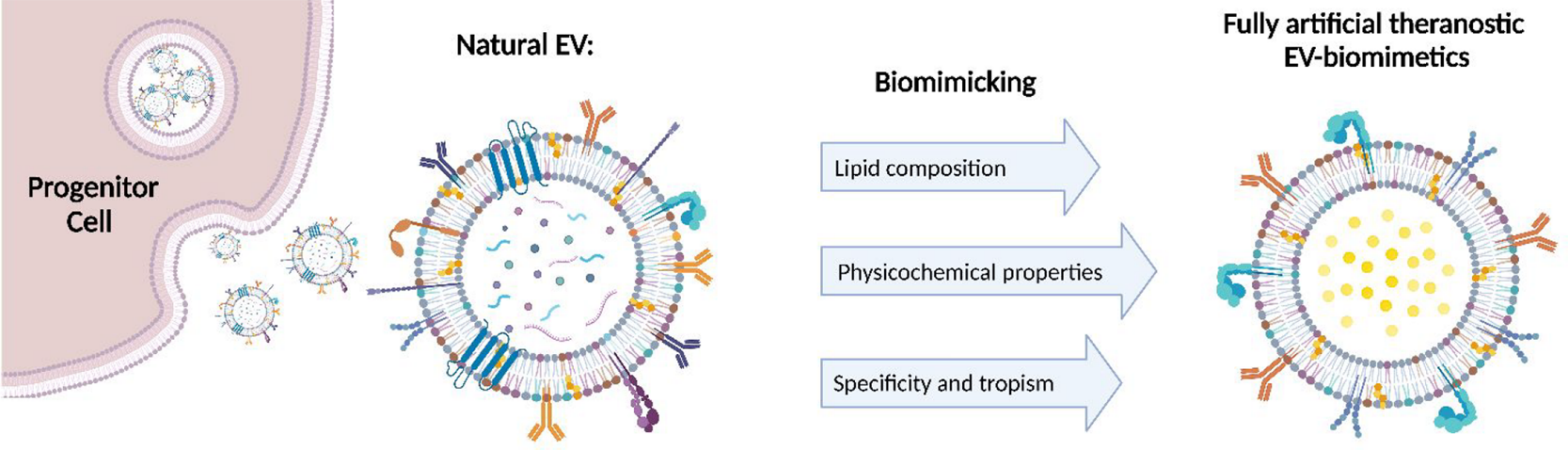

Extracellular Vesicles (EVs) are the protagonists in cell communication and membrane trafficking, being responsible for the delivery of innumerable biomolecules and signaling moieties. At the moment, they are of paramount interest to researchers, as they naturally show incredibly high efficiency and specificity in delivering their cargo. For these reasons, EVs are employed or inspire the development of nanosized therapeutic delivery systems. In this Perspective, we propose an innovative strategy for the rational design of EV-mimicking vesicles (EV-biomimetics) for theranostic scopes. We first report on the current state-of-the-art use of EVs and their byproducts, such as surface-engineered EVs and EV-hybrids, having an artificial cargo (drug molecule, genetic content, nanoparticles, or dye incorporated in their lumen). Thereafter, we report on the new emerging field of EV-mimicking vesicles for theranostic scopes. We introduce an approach to prepare new, fully artificial EV-biomimetics, with particular attention to maintaining the natural reference lipidic composition. We overview those studies investigating natural EV membranes and the possible strategies to identify key proteins involved in site-selective natural homing, typical of EVs, and their cargo transfer to recipient cells. We propose the use also of molecular simulations, in particular of machine learning models, to approach the problem of lipid organization and self-assembly in natural EVs. We also discuss the beneficial feedback that could emerge combining the experimental tests with atomistic and molecular simulations when designing an EV-biomimetics lipid bilayer. The expectations from both research and industrial fields on fully artificial EV-biomimetics, having the same key functions of natural ones plus new diagnostic or therapeutic functions, could be enormous, as they can greatly expand the nanomedicine applications and guarantee on-demand and scalable production, off-the-shelf storage, high reproducibility of morphological and functional properties, and compliance with regulatory standards.

## Introduction

Extracellular Vesicles (EVs) are lipid-based structures naturally produced by cells and secreted in the extracellular space through exocytosis.^1,2^ In the last years, a high number of studies evidenced that EVs play a fundamental role in cell communication and membrane trafficking, being responsible for the delivery of innumerable biomolecules and signaling moieties, e.g., mRNA, micro RNA, proteins, and lipids.^3,4^ Indeed, EV is a collective term including a very heterogeneous range of membranous entities such as exosomes, microvesicles, oncosomes, and apoptotic bodies, differing in size, cargo, biogenesis, release mechanism, and biological functions.^3,5−7^

EVs are composed of a lipid bilayer which is similar, but not identical, to the membrane of their respective parental cells.^8,9^ In similarity to cell membranes, EVs are a complex dynamic system made of a plethora of interacting molecules, such as phospholipids, sugars, and proteins, involved in the recognition and binding with the recipient cells.^1^ In fact, it has been demonstrated that EVs possess intrinsic tropism capabilities, i.e., the propensity to selectively head toward determined sites in the organism. In particular, cancer derived-EVs show high levels of tropism, being capable of reaching very specific tissues and delivering oncogenic messages in distant organs.^2,10−12^ EVs are emerging as key actors in tumor development and spreading, since they show the ability to promote premetastatic niche formation,^13,14^ mediate tumor invasion,^15−17^ modulate the immune response,^18−20^ and even reprogram cells, triggering or boosting their aggressiveness.^21−24^

EVs’ natural tropism,^10,25^ together with their biocompatibility,^26^ low clearance and good biodistribution,^27^ and biostability, as well as their ability to transport cargos and to cross biological barriers^28^ (e.g., blood–brain barrier^29,30^), make them a very interesting resource for theranostic scopes.^31^ For these reasons, EVs are employed or inspire the development of nanosized therapeutical delivery systems.

In fact, after 2011, when the idea to exploit EVs for targeted drug delivery was introduced, a multitude of studies have been conducted to develop EV-based delivery systems, which can be enclosed under the name of engineered EVs.^1,32,33^ The key concept of EV engineering is to isolate natural ones and to modify them, in order to produce biomimetic nanocarriers with the desired features.^33−36^ This can be achieved both indirectly, i.e., by treating the parental cells and “force” them to produce vesicles which express specific peptides or targeting molecules,^37^ or directly, by acting on EVs once they are isolated.^35^

Another important followed approach is the development of hybrid vesicles, which consists in combining or fusing natural EVs with synthetic liposomes.^38−41^

Unfortunately, nowadays no EV-based therapy has been approved because the use of naturally derived EVs brings along important obstacles.^42^ First, there is an immense heterogenicity of EVs not only considering different cell sources but also regarding the same cell line, which makes it difficult to standardize the isolation and purification procedures;^32^ then, EV extraction implies complex, time-consuming, and low-efficiency processes and strongly limits the scaling-up of these techniques. Most importantly, EVs carry with them safety concerns: naturally derived EVs possess, although quite low, an immunogenic profile.^43^ Far more critical hurdles are present when considering tumor-derived EVs. Very little is known about the key biological factors underlying tumor-associated EVs and the mechanisms that involve EVs in cancer progression and spreading.^44−47^ For this reason, although very inviting for their marked tropism, it is not completely safe to employ cancer-derived EVs or their byproducts.^1,32,33^

**Figure 1:**
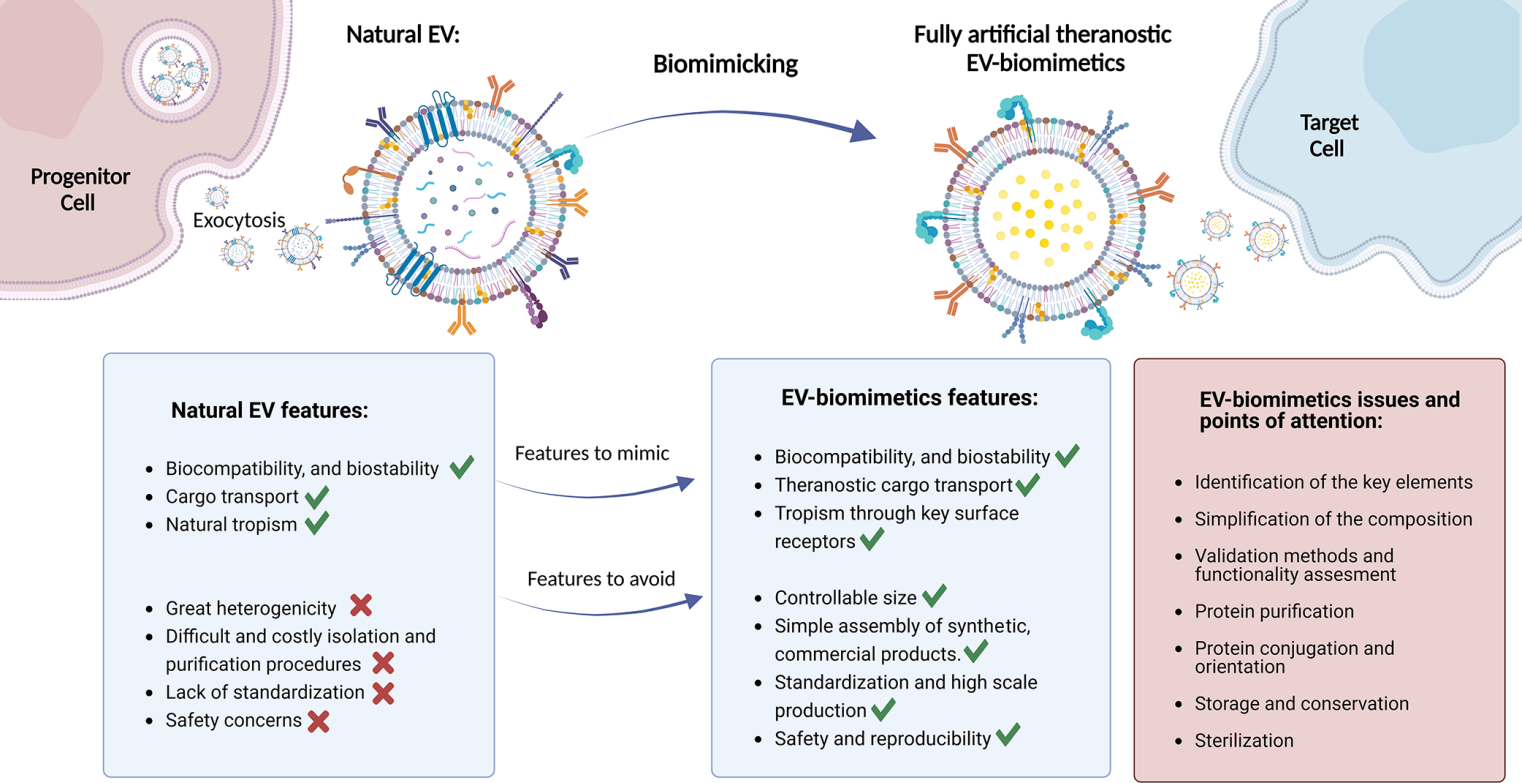
Schematic representation of the advantages and issues of EV biomimetics obtained through the bottom-up approach. Created with BioRender.com.

To overcome these obstacles, a broad variety of alternative solutions have been presented and recently reviewed by Villata et al.^33^ In brief, two branches of approaches have been developed: top-down methods consisting of disrupting cell membranes into small sections, which autonomously reassemble into nano- or microvesicles;^48,49^ bottom-up methods, on the other hand, consisting of combining molecular components such as synthetic lipids, using them as building blocks to obtain artificial lipid bilayers (liposomes) mimicking EVs, as proposed by Kooijmans et al.,^50^ or cellular membranes, as in the work of Zinger et al.^51^ It is our belief that the bottom-up approach, nowadays still in its infancy, could offer enormous opportunities, as described in Figure 1: EV biomimetics aim at the development of fully synthetic products much less complex in composition but still resembling their natural counterparts in terms of cargo delivery efficacy and homing capability. The hypothesis at the base of this approach is, in fact, that not all the components are fundamental to achieve the same functionality of the natural EVs, but it is necessary to identify the crucial ones involved in the forefront of the biological activity intended to mimic.^50^ Large-scale production of EVs is subject to the difficult, time-consuming, and costly practices in EV isolation and purification at standardized and suitable clinical grade levels. Furthermore, it is even more challenging to isolate a particular subpopulation of EVs with specific size and molecular features that can be used for therapeutic or diagnostic applications. Consequently, the development of fully artificial EV-biomimetics, showing optimal and controllable size, targeting, and cargo transfer, as well as standardized high-scale production, could effectively overcome the lack of the natural EVs of standard and clear characterization, ensuring reproducibility and safety from a pharmaceutical point of view.

On the other hand, the bottom-up approach is undoubtedly an ambitious and very challenging approach: first, it requires detailed knowledge and understanding of the nature and the function of the majority of natural EVs components to identify the fundamental ones in the extraordinary vast array of possibilities. Indeed, the biggest limitation of the biomimicking trough bottom-up approach is dictated by its nature: the complexity of the artificial vesicles will always be lower than their biological counterpart, which may cause the risk to oversimplify the system, losing the desired properties, for example, in terms of biocompatibility and delivery efficacy.

Concerning the biological activity of EV biomimetics, numerous other issues are raised when considering the combination of the chosen molecular elements, such as the conjugation of surface proteins. It is in fact fundamental to find a method which allows binding proteins to assure their purification, correct orientation, and consequently their functionality; subsequently, it is necessary to formulate valid methods to assess this biological functionality and to compare it to the natural reference one.

In the prospect of employing EV-biomimetics in vitro and especially in vivo, once repeatability and the safety of the EV biomimetic product are demonstrated, various important points of attention (which are common to all drug delivery systems) should be considered: the assessment of the stability of the system, the identification of the optimal storage conditions and long-term conservation, and the development of efficient sterilization strategies.

In this perspective, we propose the emerging field of EV-biomimetics for theranostic scopes. We introduce a possible approach to prepare new, fully artificial EV-biomimetics, with particular attention to maintaining the natural reference lipidic composition and with a consideration of the protein signature. Then, we discuss the beneficial feedbacks that could emerge combining the experimental tests with atomistic and molecular simulations of the EV-biomimetics lipid bilayer. Finally, we propose the use of common characterization methods to validate the behaviors of EV-biomimetics, effectively mimicking the natural ones.

## A Novel, Practical Strategy

It is from this EV biomimetics approach that a novel strategy can be considered: the mimicking of natural EVs in terms of lipid composition. In fact, in developing artificial EVs, great effort is typically directed into the surface protein profile or nucleic acids, because of their fundamental role in communication and interaction with cells.^52^ The lipidic profile, instead, generally fades into the background, recognizing in lipids only a structural role. Nonetheless, lipids are emerging as essential elements, actively involved in the biological functions of EVs.^33,52^ Actually, the number of studies investigating natural EVs membranes is rapidly increasing, further corroborating the relevance of the lipidic composition.^18−21^ To the best of our knowledge, there are very few publications concerning the production and characterization of EV biomimetics, and there is certainly space for further investigations and improvement. Sakai-Kato et al.,^53^ for example, presented a very interesting study of different formulations mimicking the exosomes secreted by HepG2 cells in terms of physiochemical properties and a representative lipid composition. The lipid formulations are though very simple, including a maximum of four components and devoted principally to evaluate the differences between formulations, varying among different types of saturated fatty acids and their unsaturated counterparts (i.e., DSPS versus DOPS). Lu et al.,^54^ on the other hand, performed a comparison between conventional liposomes and exosome-mimicking liposomes, which were prepared by mixing DOPC/SM/Chol/DOPS/DOPE at a molar ratio of 21/17.5/30/14/17.5. The so-called EXO shows a more complex and complete composition than the ones proposed by Sakai-Kato, but they aim to reproduce a “generic” lipidic formulation, a “synthesis” among different EV populations coming from different cell lines, through the identification of common traits or trends. Although this is a very promising approach, capable of reproducing a “universal” EV mimic, in this way, specificity and tropism ability of a particular EV population may be lost. Furthermore, the study is completely focused on reproducing the lipid composition in terms of phospholipid categories, which are determined by the nature of the polar head of the molecule, and no attention is given to the fatty acid population. In natural EVs, actually, every single phospholipid type (e.g., Phosphatidylserine (PS) or Phosphatidylcholine (PC)) is present with a very broad distribution of fatty acids, which can be shorter or longer carbon chains, saturated, monounsaturated, or polyunsaturated. These hydrophobic tales strongly influence the physiochemical characteristics of the lipid double layer such as fluidity, viscosity, and rigidity and are essential to the formation of lipid domains or rafts, influencing the way the hydrophilic heads move and group together and expose their functional groups or other anchored signaling moieties.^55,56^

**Figure 2:**
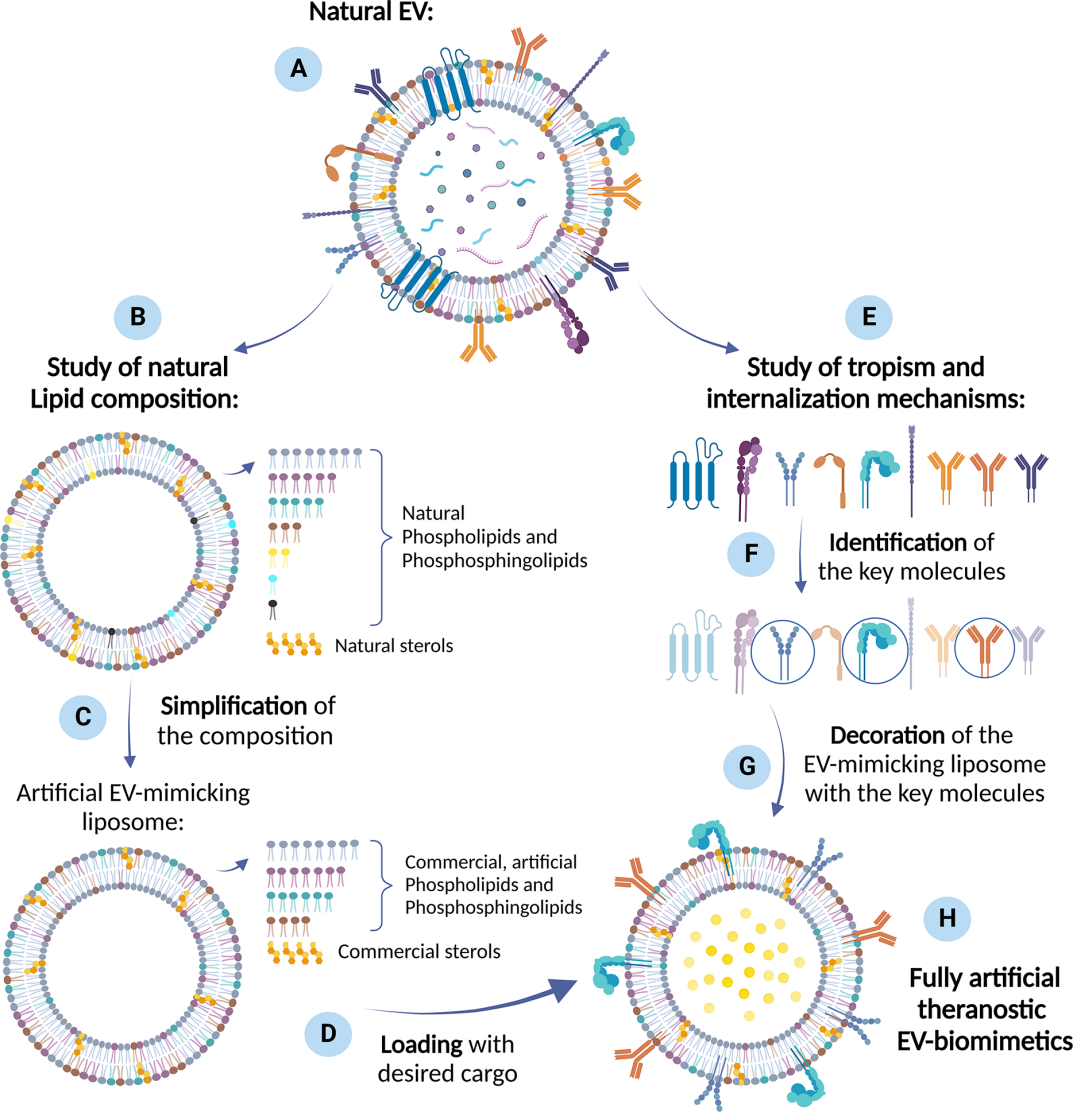
Practical approach to develop EV biomimetics. The starting point is the choice of (A) a natural EV population with the desired characteristics and homing capabilities. (B) Lipidomic studies of natural EVs provide a reference composition, from which simplifications can be introduced. (C) These simplifications may consist in identifying the most abundant lipid components and excluding the less abundant ones, balancing the amount of the different lipid categories, while maintaining the correct ratios among the different types of fatty acids. In this way, artificial EV-mimicking liposomes can be produced and (D) loaded with a cargo (e.g., drugs, genetic materials, nanoparticles, proteins) through consolidated methods from literature. (E) The study of the biological mechanisms behind a specific tropism ability, can lead to the (F) identification of key molecules enabling homing attitude toward specific target cell receptor. (G) The decoration of the cargo-loaded, artificial EV-mimicking liposome with the designated proteins or peptides concludes the process to produce (H) fully artificial theranostic EV biomimetics. Created with BioRender.com.

In view of the aforementioned premises, it is therefore possible to conceive a new way of creating EV biomimetics (Figure 2). As a practical approach, the starting point could be the lipidomic studies of well-known cell lines (in particular, cancer cell lines) and the produced EVs, which show the desired homing capabilities. Since the type and number of lipidic components in the EVs are broad, simplifications can be introduced. It could be possible, for example, to identify the most abundant lipid components of such natural EVs and trace a simplified composition, grouping phospholipids of similar chemical features, charge of the polar head, with particular attention to the presence of saturated and unsaturated bonds in the hydrophobic tails. The ideal would be reaching a formulation which balances the amount of the different lipid categories (discarding the less abundant ones), while maintaining the correct ratios among the different types of fatty acids (saturated or mono- or polyunsaturated). In fact, it would be interesting to include in the lipid formulations also polyunsaturated fatty acids and explore their effects in terms of physiochemical properties and biological activity of the synthetic EVs.

It is then foreseen to load the artificial EVs with active compounds, enabling an advanced function in terms of imaging reporter, diagnostic, or therapeutic activities. Examples of these various functions can derive from the already-reported literature concerning both natural EVs or liposomes, proposing the incorporation of drugs,^57,39^ genetic materials,^58^ organic dyes, aptamers,^59^ peptides, and more in general proteins ranging from enzyme to antibodies,^60^ and even solid-state nanoparticles of various nature, size, porosities, properties, and functions.^61−65^

In order to complete the so-obtained synthetic EV and optimize its cargo-delivery efficiency, the following step would be the identification of key proteins enabling homing attitude toward the specific target cell receptor and the decoration of the synthetic EV surface with the designated proteins or peptides. To do this, we evidenced literature studies investigating natural EV membrane proteins to unravel their innate biological mechanisms. Among these studies, we distinguish between (i) strategies to identify proteins involved in homing; (ii) strategies to identify proteins involved in cargo transfer. In this way, it could be in principle possible to produce fully artificial vesicles, mimicking the reference natural EVs and conferring them the desired biomimetic and specific targeting properties in a controllable and reproducible manner.

Proteins are of top-interest in the context of EVs research, and many literature references are available, showing the identification of surface proteins at the EV membranes able to mediate the EVs interaction with recipient cell and internalization. For example, to find the key factors of the well-known metastatic organotropism of certain cancer cell lines, Hoshino and colleagues^12^ followed an approach based on proteome profiling of tumor-derived exosomes, joined with a biodistribution analysis. Remarkably, they not only found that exosomes are capable of fusing with cells of the same metastatic target of their progenitor cells but also that integrins are responsible of organ-specific uptake (in particular, ITGβ_4_ and ITGβ_5_ promote lung and liver metastasis, respectively). Therefore, this study demonstrated that it is possible to define a specific set of proteins expressed by EVs (distinct to the one expressed by their parental cells), which dictates the EV adhesion to particular tissues and ligands of their specific extracellular matrix.

Tetraspanins, integrins, and immunoglobulins have been reported to take part in EV internalization, mediating their fusion with plasmatic or endosomal membrane, as well as other mechanisms, such as micropinocytosis, phagocytosis, and clathrin-mediated endocytosis. In this perspective, the roles of specific protein–protein interactions and of lipid rafts were investigated and challenged by further adding specific antibodies to the EV surface or of chemical inhibitors to the cell receptor surface able to interfere with a specific uptake paths or time of the interaction. A recent study devoted to identifying the homing capability in healthy cell-derived EVs was reported by Limongi et al.^60^ The authors evaluated the intercellular trafficking of B lymphocyte derived EVs and their homing capability toward their parental cell line and toward two hematological cancer cells, a lymphoid cell line and a myeloid one. Data showed interesting tropism toward the parental cell line but also to the lymphoid human cancer cell line, with significantly less internalization toward the myeloid cell line. To challenge this natural homing capability, authors added a monoclonal antibody (anti-CD20) on the EV surface and demonstrated the ability to produce a selective targeting directed toward the lymphoid cancer cell line, which overexpresses the CD20 antigen. This EV-engineering with additional proteins clearly shows that it is possible to tune the innate EV tropism, at least in vitro.

Concerning the strategies to identify proteins involved in cargo transfer, the literature mainly reports data on tumor-derived EVs^10^ which are supposed to circulate in the bloodstream and promote metastasis to specific target tissues, releasing their content to the recipient cell. So far, many molecular mediators (phosphoproteins, tetraspanins, integrins, lectins, proteoglycans, fibronectin, laminin, and phosphatidylserine, to cite some) at EV surfaces have been identified as possibly participating in this target cell docking, as well as in EV uptake, downstream signaling, and processing in recipient cells.^66^ Thereafter once the recipient cells have been reached by the cancer-derived EVs, their genetic material content, i.e., various types of RNA, is released, triggering both phenotypic and molecular reprogramming of the recipient cells and more in general inducing the multiple steps of metastasis formation, i.e. premetastatic niche formation^67^ in the liver and lung, vascular remodeling, cell migration to metastatic site, immune evasion,^68,69^ and even therapy resistance.^22^ The relationships among these processes and the EV molecular components, i.e., surface proteins and RNA content, are currently under study and still have to be deeply understood. EVs produced by colorectal cancer have been identified as being enriched with β-like 1 integrins and to activate fibroblasts of remote organs, like liver and lung, and promote the formation of a premetastatic niche.^70^

## A Step Forward

To allow the conception of EV biomimetics molecular simulations can also be used. Actually, molecular simulations have also started to approach the challenging task of modeling lipid organization in a bilayer structure and their self-assembly. To do this, lipids can be considered as supramolecular structures with coarse-grained (CG) force field models. In this perspective, machine learning approaches have been recently considered with the aim to accelerate the development of accurate coarse-grained molecular models.^71^ It is of prominent importance, in the development these molecular systems, to avail on experimental data (like geometric parameters such as area per lipid and/or bilayer thickness) and of reliable all-atom force fields. Examples were reported demonstrating the ability of advanced CG models to simulate lipid–lipid interaction, self-assembly into lipid bilayer, formation of vesicles, and vesicle fusion using different model lipids.^71−73^ These results, although preliminary applied to a subclass of lipids, i.e., phosphatidylcholines (PC), can be transferrable to a broader plethora of phospholipids and other classes of macromolecules for which reference experimental or simulation data are available in order to properly train the developed algorithm.

Such simulations have certainly the potential to enormously support the development of artificial EV-biomimetics: experimental tests can be combined with atomistic and coarse-grained simulations, able to predict the physical characteristics of interest and the dynamics of the lipid bilayer (i.e., the formation of lipid rafts or different phase domains). With reciprocal feedback, it is possible to fully optimize the EV biomimetics, tailoring and adjusting on one side simulation parameters and on the other one the lipidic composition according to the desired physiochemical characteristics (which more resemble the natural ones).

Validation methods should then be implemented to verify that the behavior of the produced artificial EVs is effectively approaching that of to natural ones or, in the best-case scenario, overcoming the expectations. We refer here in particular not only to the targeting and cargo transfer behavior of natural EVs but also to their biostability, narrow size distribution, and cargo transport. Physical–chemical characterizations of the size, ζ-potential, structure, and viscosity would constitute the preliminary characterizations. The following step is the biochemical analysis of correct incorporation and orientation of proteins, integrity and functionality of the included genes, as well as drugs or dyes. Finally, the most relevant verifications enabling such artificially conceived EVs to approach the natural one is related to the biological functions, i.e., homing capability, internalization into target cells or tissues, ability to cross biological barriers, as well as the intrinsic safety, including the absence of off-target and immunogenicity and maintenance of sterility during production.

In view of the complexity of the aforementioned validations, artificial systems at increasing levels of complexity and function can be proposed, enabling a step-by-step approach toward the development of effectively functional and completely artificial EVs.

## Conclusions and Future Perspectives

In recent years, in response to the request of medicine to nanovehicular drugs or active compounds and improve the quality of treatments in terms of specificity and efficacy, several strategies have been followed. Natural EVs and EV-based delivery systems have been explored, but several limitations preclude their employment, including high costs, difficulties in EV isolation and purification, and the lack of standardization and reproducibility. As a future direction, we suggest the development of fully artificial vesicles, through a bottom-up approach, which aims in the first instance to find a simplified liposomal formulation, mimicking the natural EV composition of a well-known EV population. The first focus of the research would be then directed toward in-depth lipidomic studies and the identification of the key elements, which will allow the simplification step. A step-by step approach could be applied, allowing the investigation of lipid formulations with increasing complexity and the exploration of the effect of different fatty acids (including different types and ratios of unsaturated ones) on the characteristics and the dynamics of the lipid bilayer. To fully optimize the physiochemical properties of such EV-mimicking liposomes, we have individuated an interdisciplinary approach between experimental data and atomistic and coarse-grained models as a possible way. Since the first rudimentary lipid-layer models have recently been developed, we foresee that they will rapidly advance, becoming more and more accurate, rendering themselves a pivotal tool in the prediction of the EV-biomimetics physical properties and behavior.

Further research is needed in the proteomics field, in order to understand and select which are the key molecules, exposed by the natural EVs, responsible for their strong tropism and cargo transfer. The ability to selectively head toward a precise site in the human body and being taken up by a particular cell type is the most desired specification proper of natural EVs. With the deep understanding of biological mechanisms involved in natural tropism, the decoration of artificial EVs with a simplified version, i.e., a subset, of proteins or peptides and the use of specific phospholipids formulations, it is in principle possible to achieve the same homing capability and cargo transfer of natural EVs.

In this sense, building artificial EVs strategies can be implemented to use native protein or recombinant ones, as well as using native versus synthetic lipids. Despite the possible cost of the single component, the abundance of natural versus recombinant proteins or of natural vs artificial lipids and their isolation and purification protocols are a matter of thought. Furthermore, after identifying the key proteins to be incorporated in the artificial EVs, the process of membrane protein integration into the lipidic shell should be carefully considered, to avoid denaturation, alterations, or wrong orientations of active sites. Finally, concerning the lipidic mixture, the packing parameter of each lipid type should be considered, as well as which position will preferentially occupy each lipid type, i.e., the inner or outer leaflet of the bilayer or if they will distribute uniformly throughout the lipid shell. Furthermore, to allow target cell recognition and cargo transfer, the surface mobility of targeting proteins has to be reproduced as in natural EVs, guaranteeing an appropriate lipid bilayer fluidity and correct lipid reorganization and shuffling. These dynamics will affect the functional behavior of the synthetic vesicles and are all obviously hot topics of multidisciplinary discussions, at the interface of chemistry, physics, molecular dynamics, simulations, and biology.

It is clear that expectations from both research and industrial fields could be enormous, as successful artificial EVs having the same key functions of natural ones plus new diagnostic or therapeutic functions can enormously expand the nanomedicine applications and guarantee on-demand and scalable production, off-the-shelf storage, high reproducibility of morphological and functional properties, and compliance with regulatory standards.

## Key Concepts

It is our belief that nowadays developing EV-biomimetics through bottom-up approach is fundamental, since the process toward clinical application is easier and safer for such fully artificial, controllable products than the application of natural EVs. Some good examples of EV-biomimetics have been already reported in the literature, but improvements and optimization are still needed: it is essential to design and create a product with the same cargo transfer and targeting features of natural EVs, but with simplified composition and functionalization, which has to be ad hoc formulated and focused for the specific case study.

## Abbreviations

EVs: extracellular vesicles
DSPS: 1,2-distearoyl-*sn*-glycero-3-phospho-l-serine
DOPS: 1,2-dioleoyl-*sn*-glycero-3-phospho-l-serine
DOPC: 1,2-dioleoyl-*sn*-glycero-3-phosphocholine
SM: sphingomyelin
Chol: cholesterol
DOPE: 1,2-dioleoyl-*sn*- glycero-3-phosphoethanolamine
PS: Phosphatidylserine
PC: Phosphatidylcholine
CG: coarse-grained
